# Supplementary material for: Identification of low threshold off-target activation pathways during stimulation of carotid baroreceptor afferents in swine
Source: bioRxiv. 2026 Jan 2:2026.01.02.697436. Preprint. [Version 1] doi: 10.64898/2026.01.02.697436 (PMC12776398; doi:10.64898/2026.01.02.697436)
Supplement: Supplement 1 [file media-1.pdf]

Supplemental Document

Methods:

Settings for Mean Arterial pressure calculation from recording blood pressure.

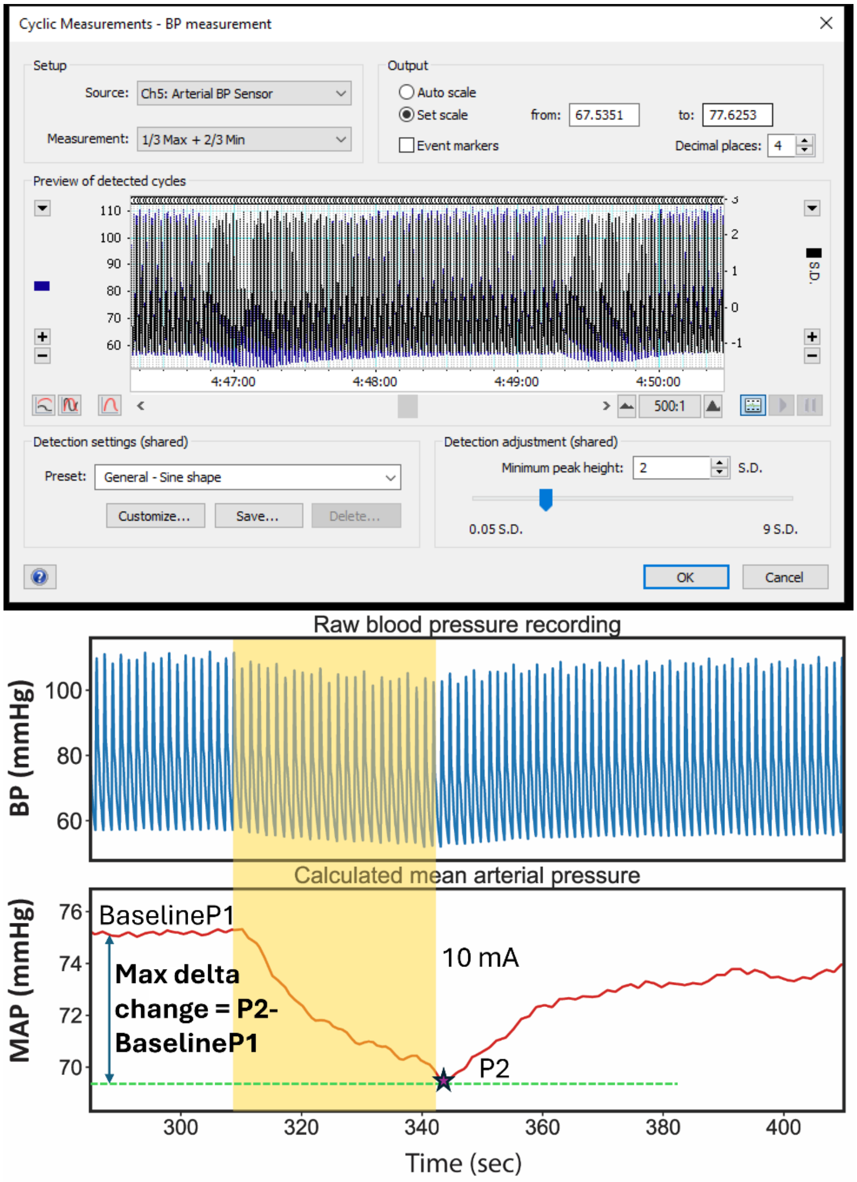

Supplemental Figure 1: Top: Settings for ADIntruments Labchart for thresholding raw blood pressure data to calculate Mean Arterial pressure. Bottom: BP response to stimulation calculated as Max delta change in MAP from baseline value.

Animal number table and stim params

Subject number	Sex	Wt	Stimulation frequency (Hz)	Stimulation pulse width per phase (usec)

Subject 1	M	35	75	350
Subject 2	F	33		
Subject 3	F	40.3	25	250
Subject 4	F	38	75	350
Subject 5	M	40.1	25	350
Subject 6	M	29.5	25	175
Subject 7	F	41	25	350
Subject 8	M	40.2	25	350
Subject 9	F	38.3	25	350
Subject 10	M	41.2	25	350
Subject 11	M	32.5	25	350
Subject 12	M	43	25	350
Subject 13	M	33.8	25	350

Supplemental Table 1: Cohort subject details for weight, sex and stimulation parameters used for pilot and functional studies. Subject 1-5 had an old blood pressure calibration technique and were not included in dose response curve analysis.

Results:

Microdissection

Subject 3

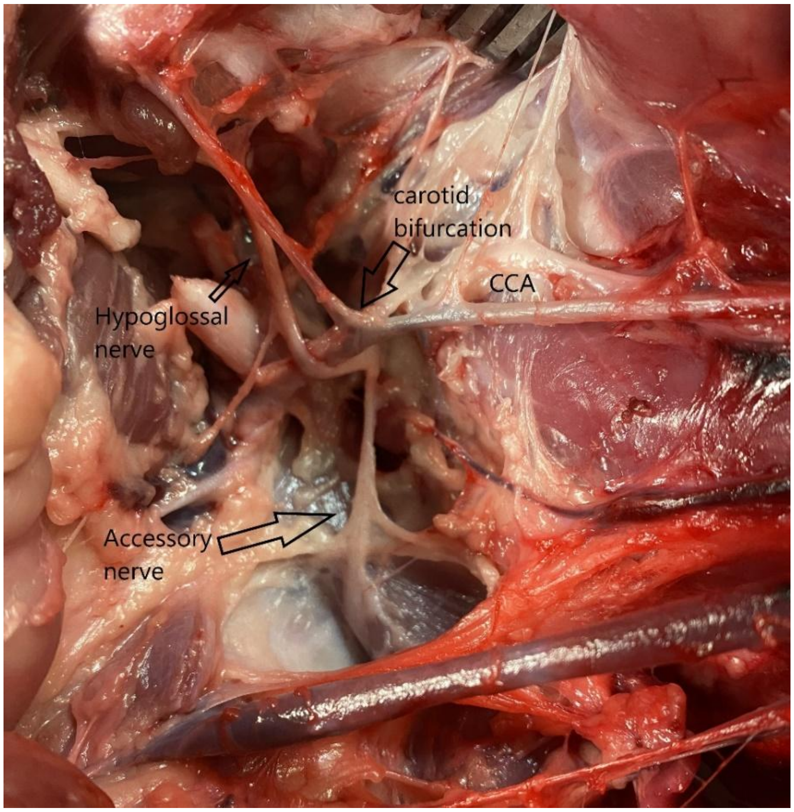

Supplemental Figure 2: Postmortem dissection to identify and trace off-target nerves hypoglossal and accessory back from their muscle groups to the carotid bifurcation. Both nerves enter the foramen dorsal to the bifurcation.

Subject 9

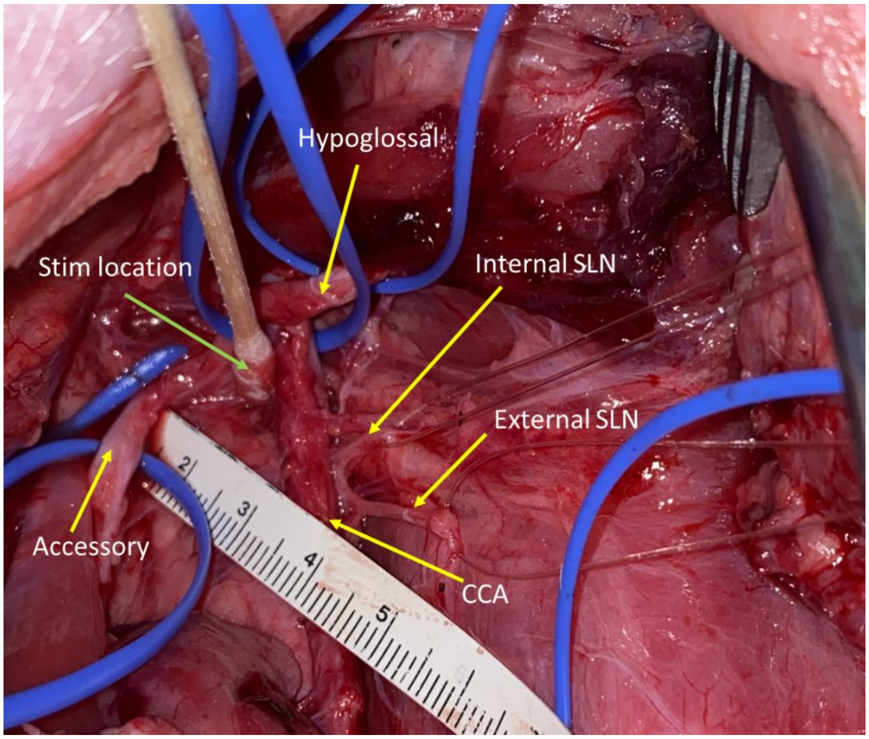

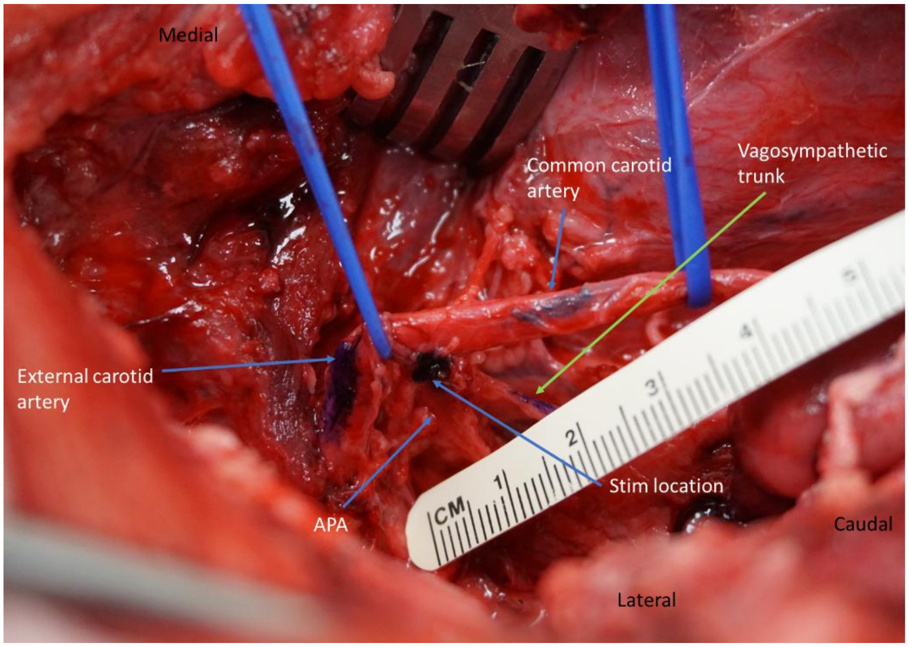

Supplemental Figure 3: Dissection showing off target nerves and their distances during stimulation (Top). (Bottom) Approximate stimulation location at the APA-OA bifurcation with the carotid artery retracted cranial and ventral.

Subject 13

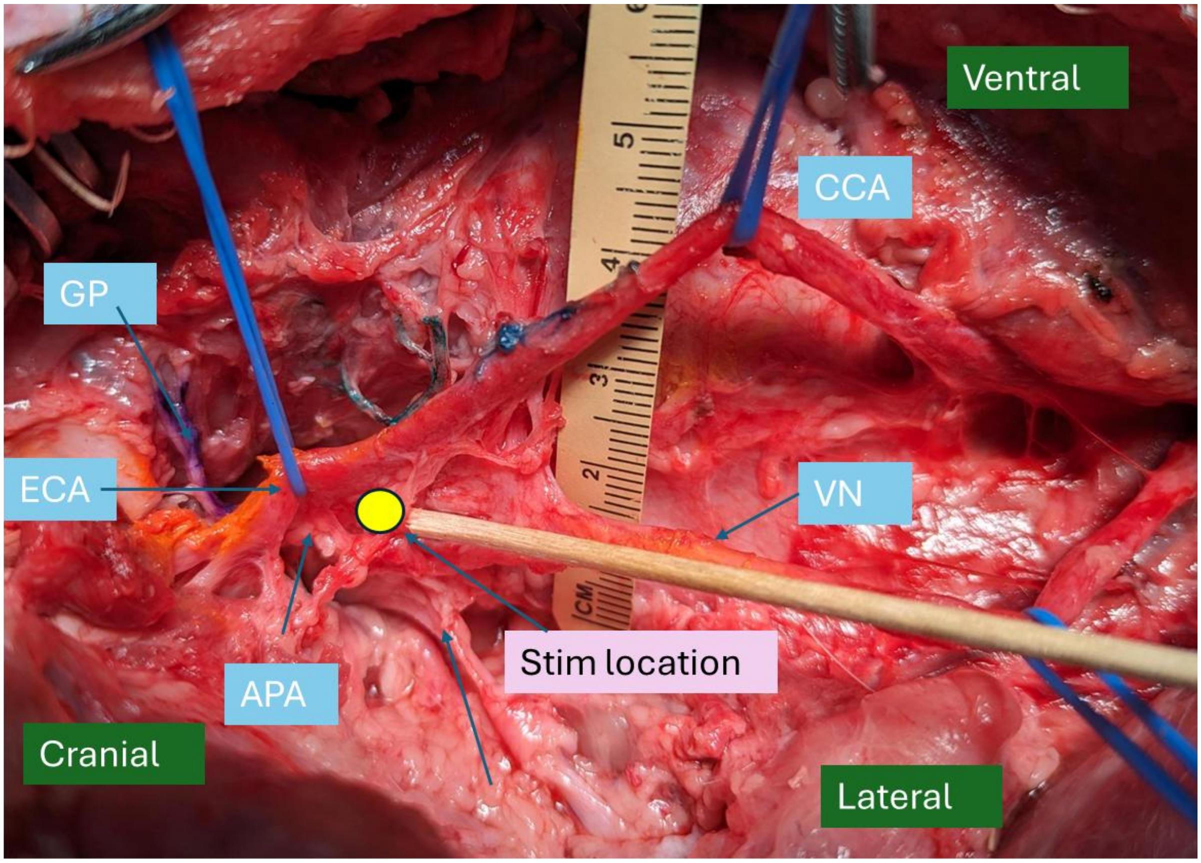

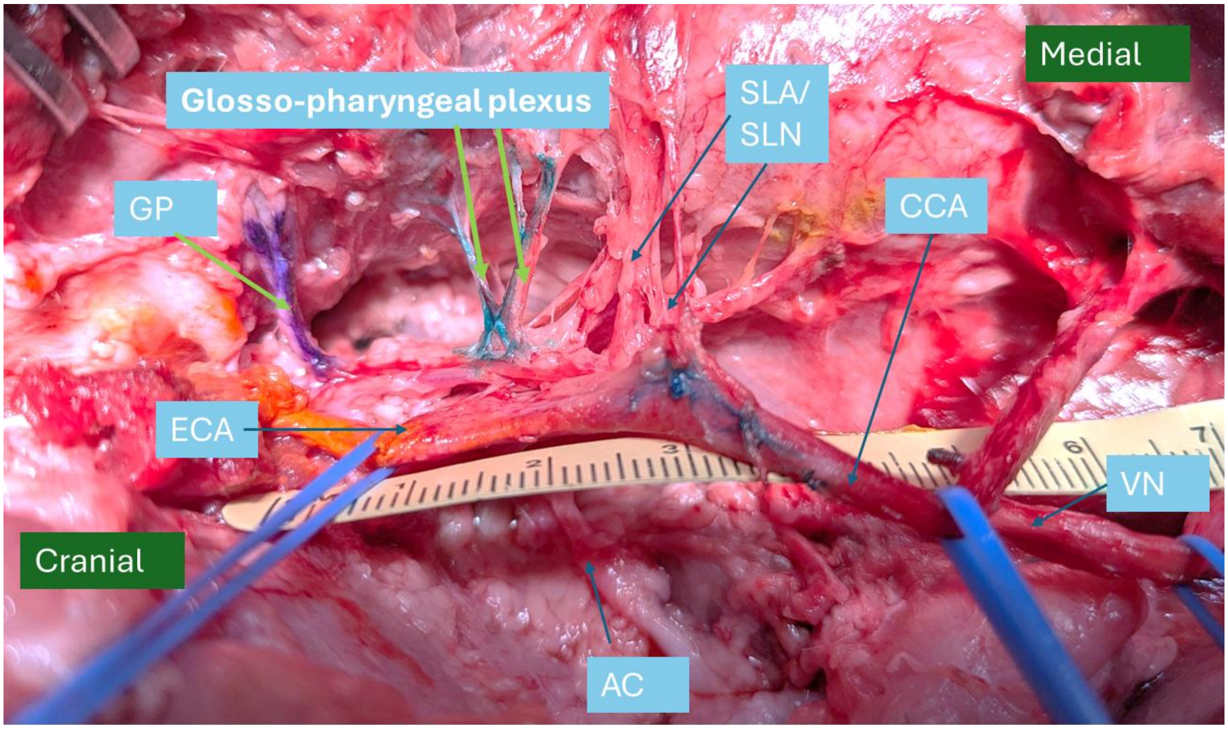

Supplemental Figure 4: Top) Post-mortem dissection showing approximate stimulation location at the APA-OA bifurcation with the carotid artery retracted medial and ventral. Bottom) Dissection showing glosso-pharyngeal plexus which innervates the constrictor muscle along with other off-target nerves. GP: glossopharyngeal nerve, AC – accessory nerve and VN vagus nerve.

Additional subjects nerve transection data

Accessory nerve

Subject 3

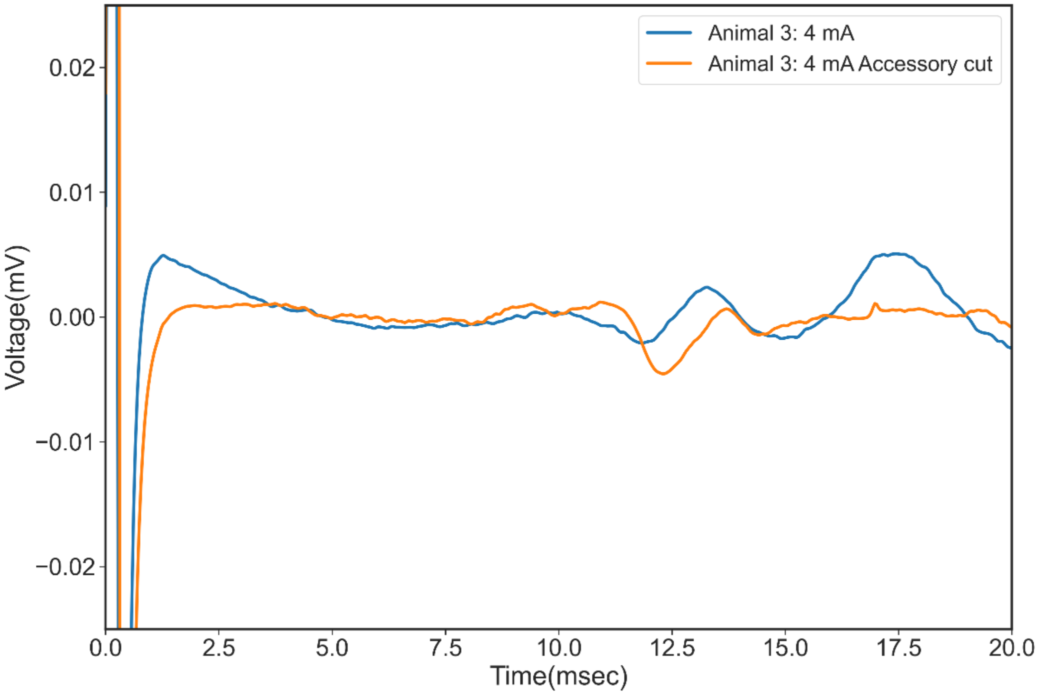

Supplemental Figure 4: Nerve transection of the only the external branch of the accessory nerve as it innervated closer to the muscle, eliminated only part of the EMG signal. This data led to a change in location of accessory nerve cut to be more cranial, medial/dorsal to the parotid gland exit point and closer to the stimulation location (as it traverses dorsal to the foramen) where the nerve is a well coalesced trunk with all its branches.

Subject 6

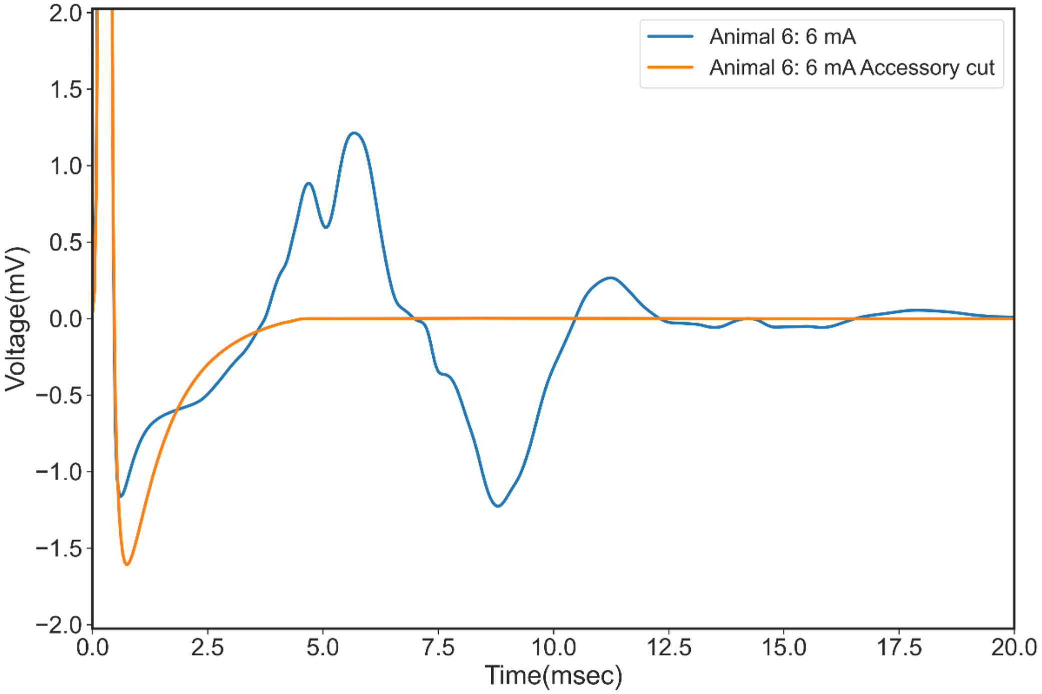

Supplemental Figure 5: Complete elimination of EMG post accessory nerve trunk transection

Subject 10

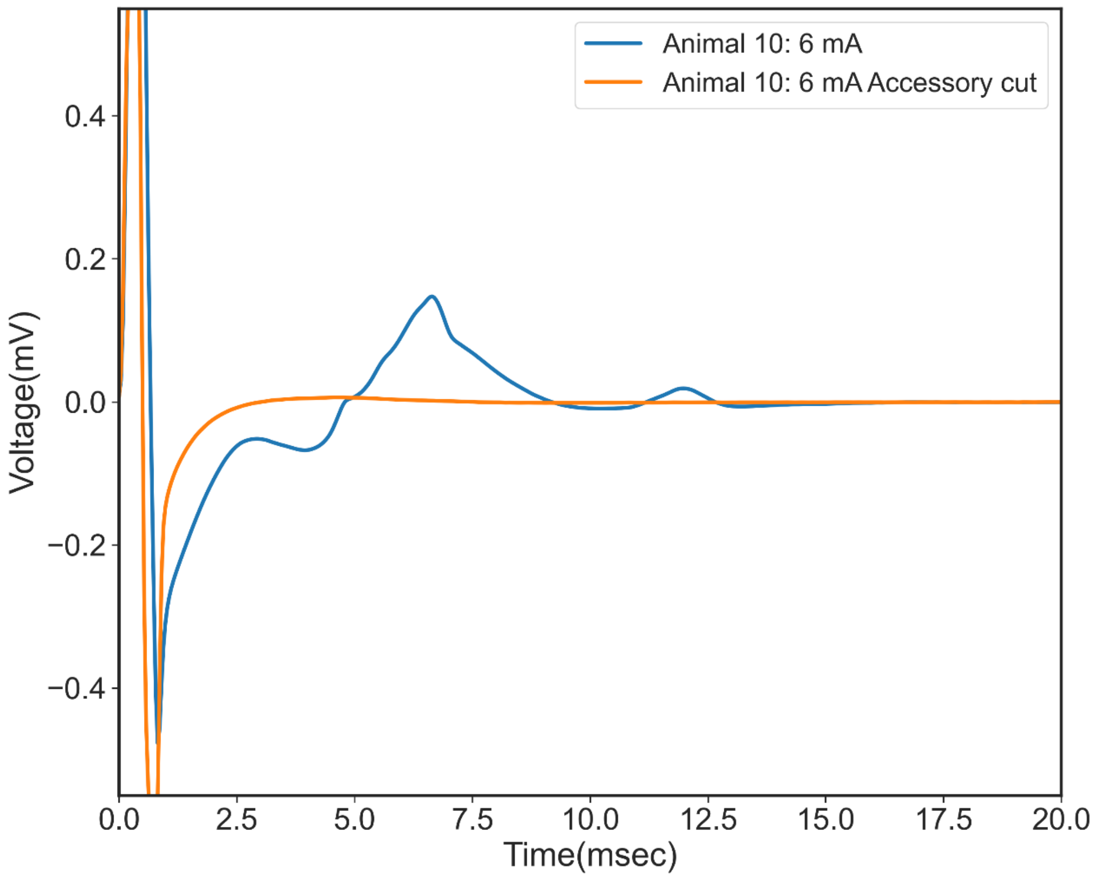

Subject 12

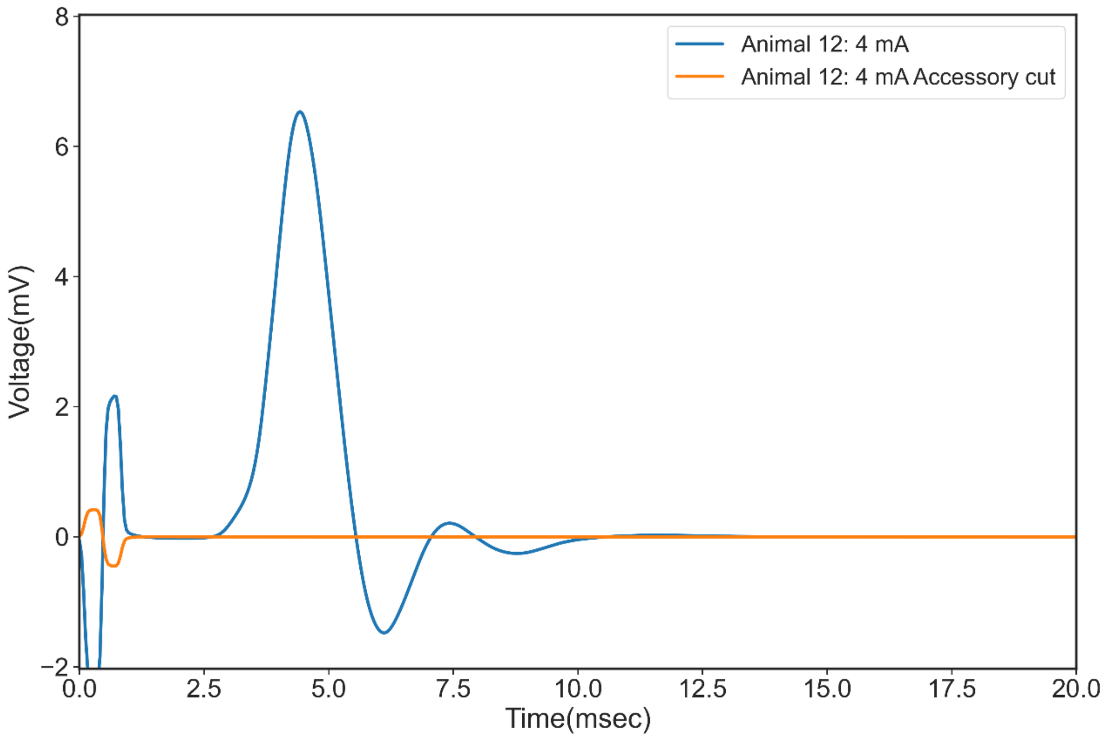

Hypoglossal

Subject 5

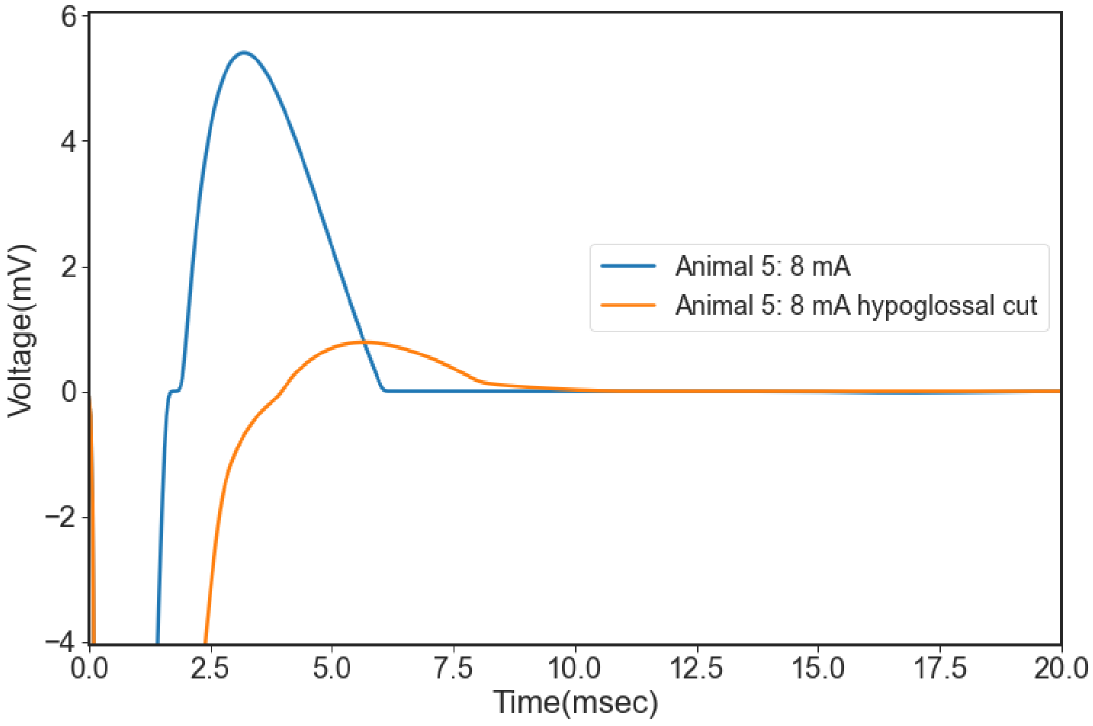

Subject 6

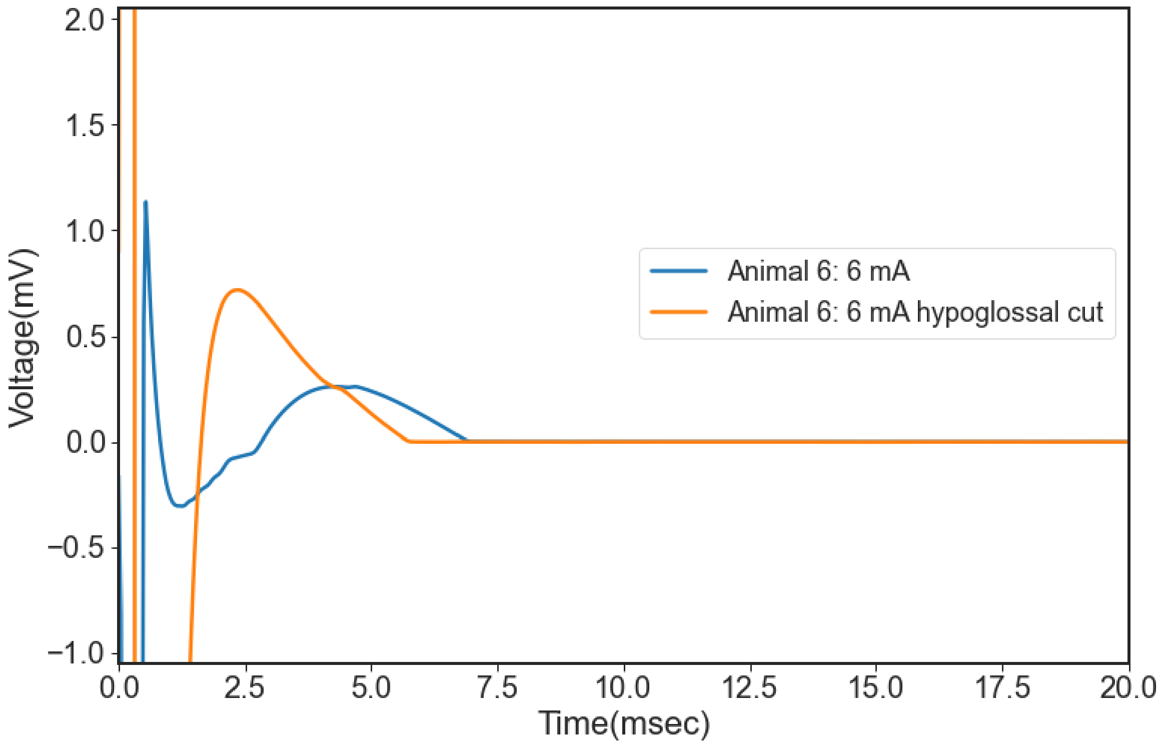

Subject 9

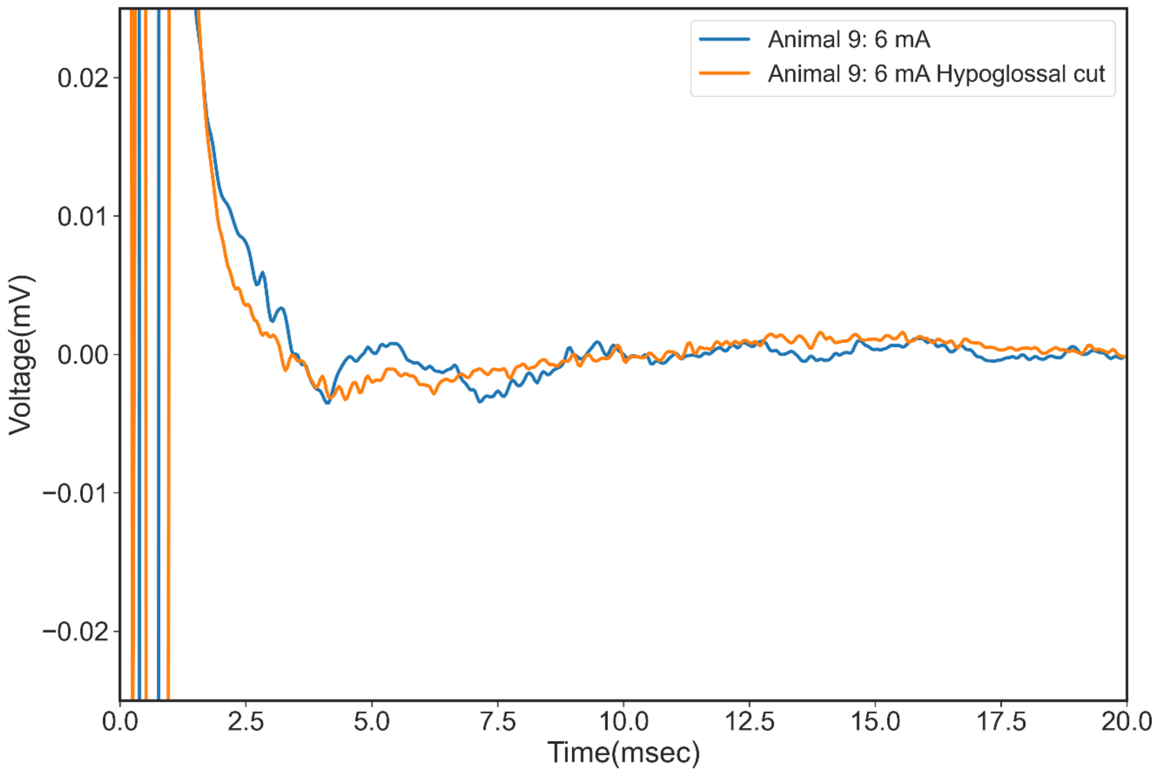

Subject 11

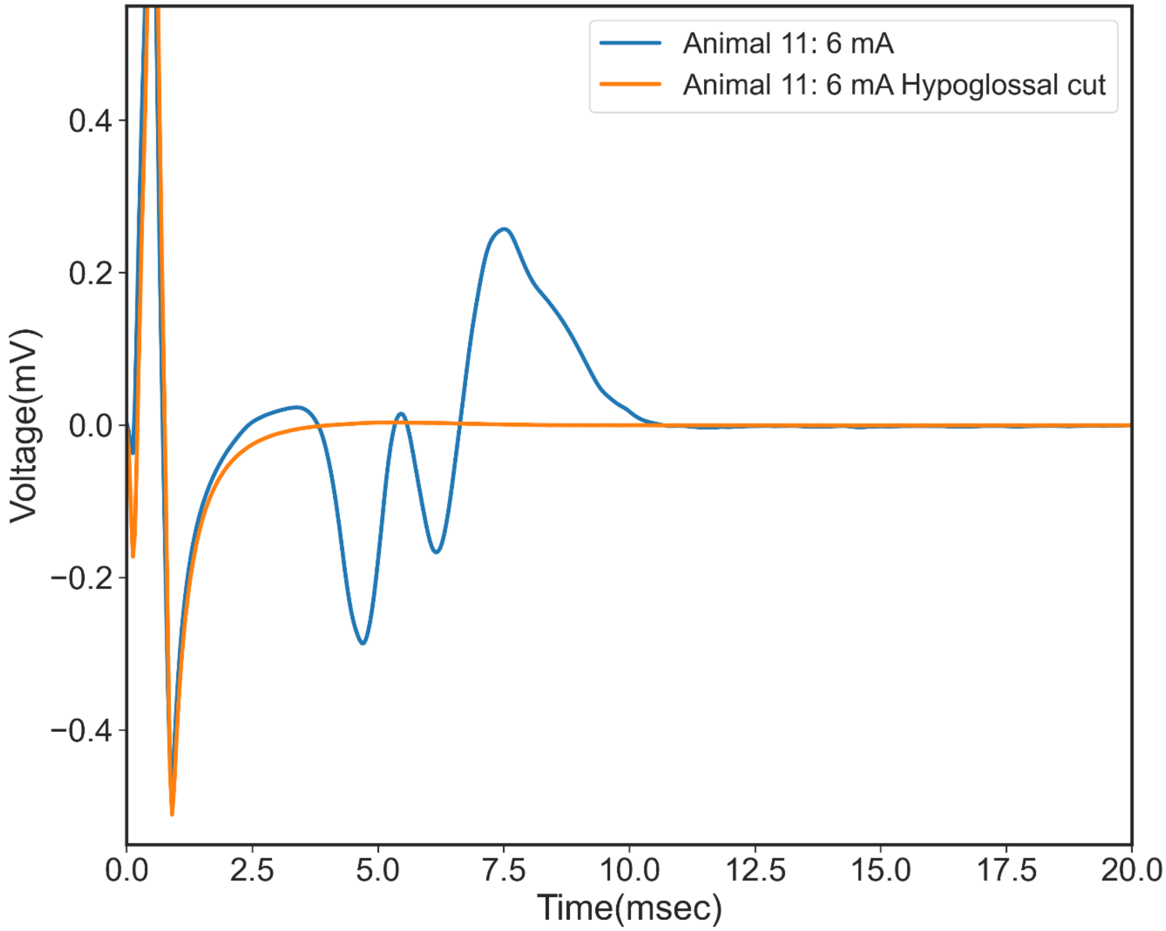

Glosso-pharyngeal plexus for Constrictors

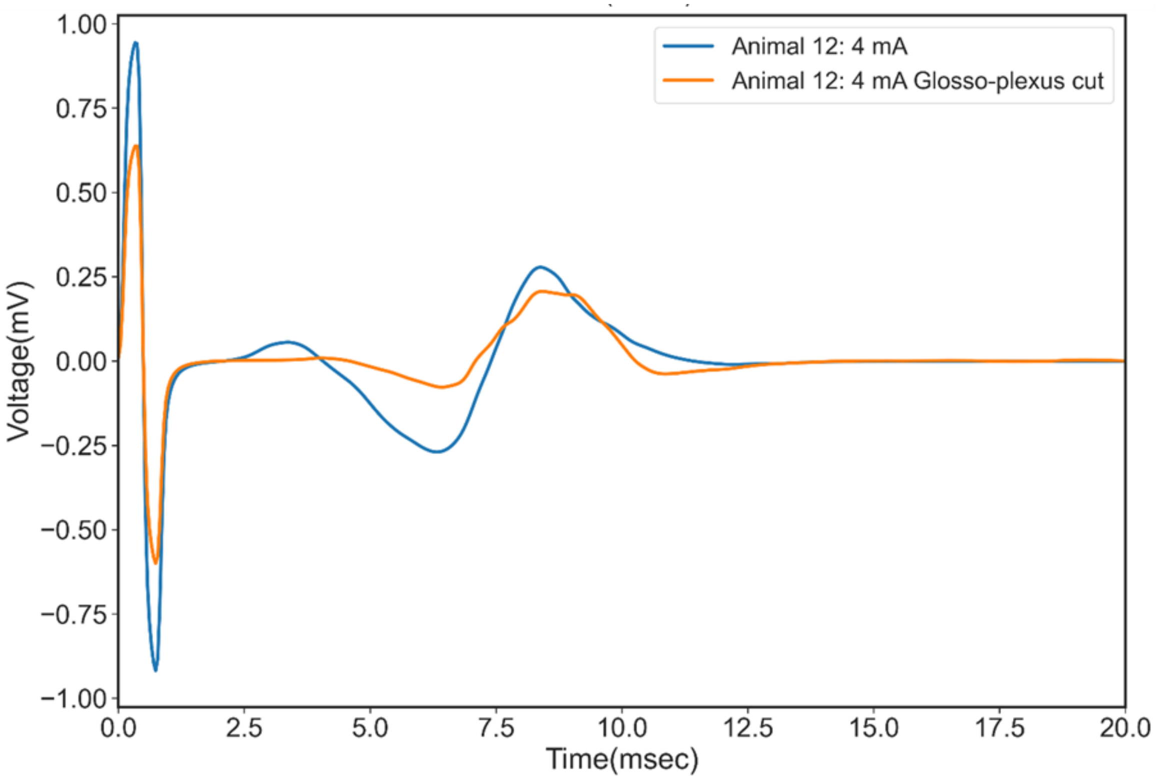

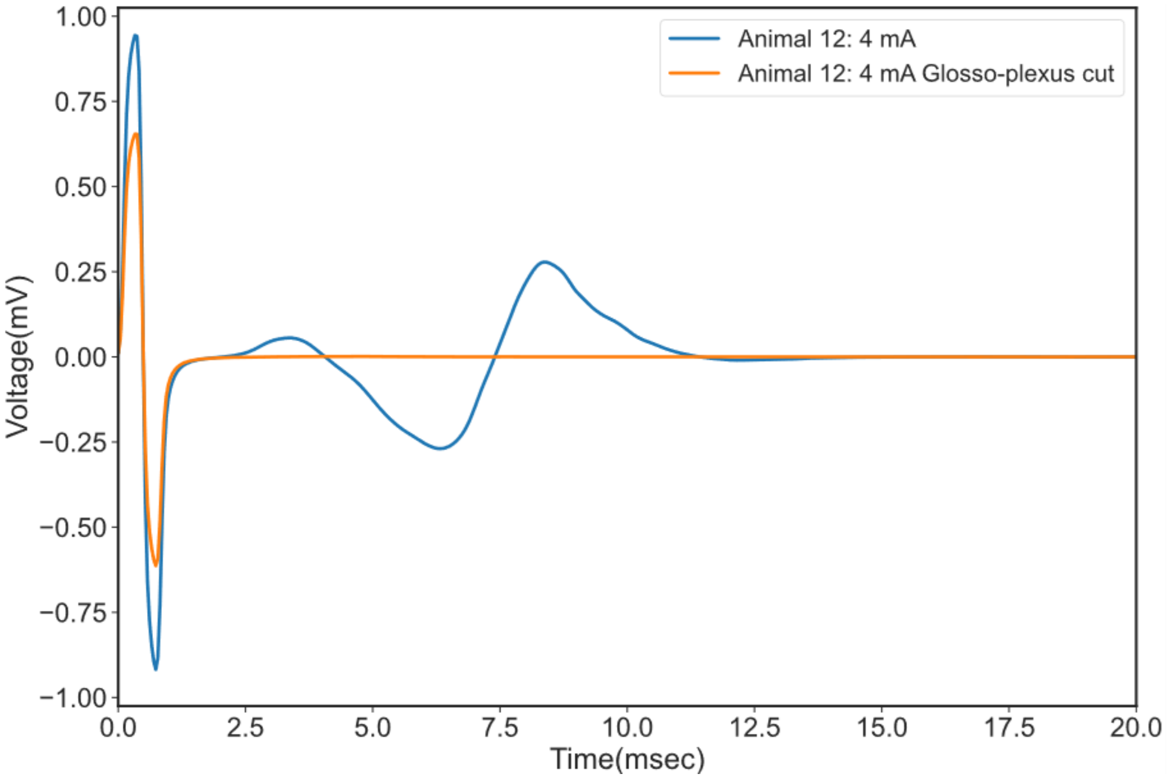

Supplemental Figure 6: Top) EMG signal recorded from the constrictor muscle group post transection of the SLN, RLN and one branch of glossopharyngeal plexus. The recorded EMG was attenuated although not eliminated. Bottom) Constrictor EMG eliminated post transections of the plexus from the glossopharyngeal and the pharyngeal branch of the vagus nerve. These data pointed to one single point of nerve activation but putatively multiple small branches innervating the same muscle.

Trapezius muscle instrumentation issues

The trapezius muscle is responsible for shoulder movement and is difficult to instrument and reliability record EMG signals from. The location and constant jostling of the wires due to movement resulted in the EMG wires being pulled out in multiple subjects. As the recording from this target muscle could not be replicated across subjects, it was dropped from the analysis and is not reported as a main off-target muscle although the accessory nerve does innervate it.

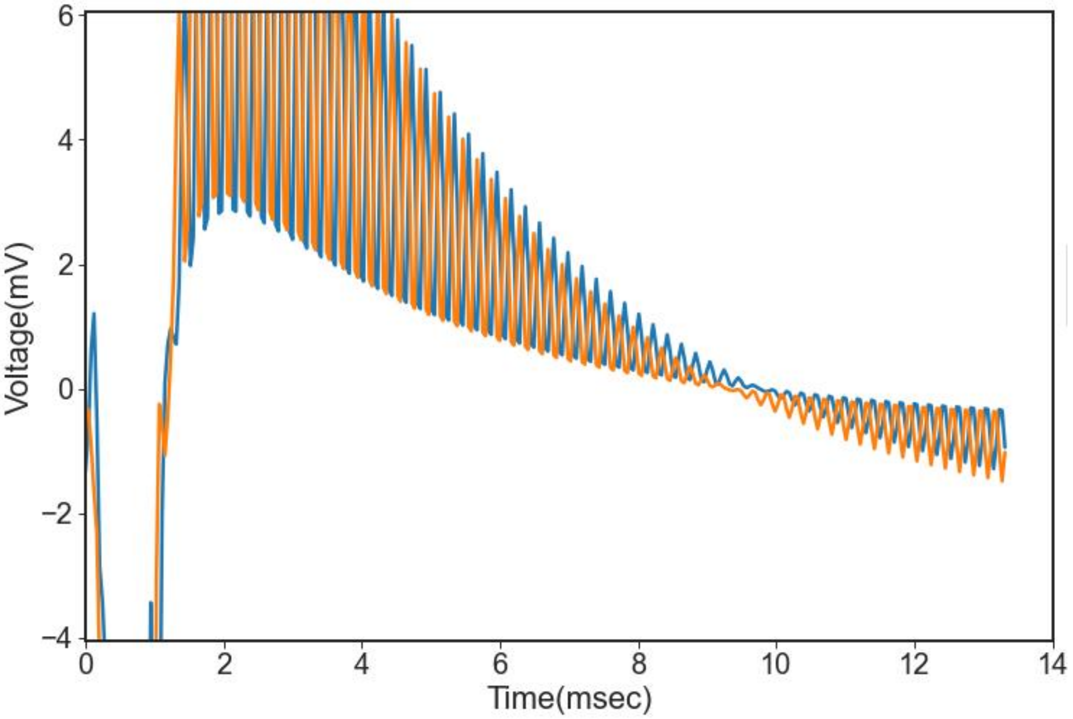

Supplemental Figure 7: Lose wires of the Trapezius EMG resulted in noise and unreliable recordings

Blood pressure drops

Subject 10

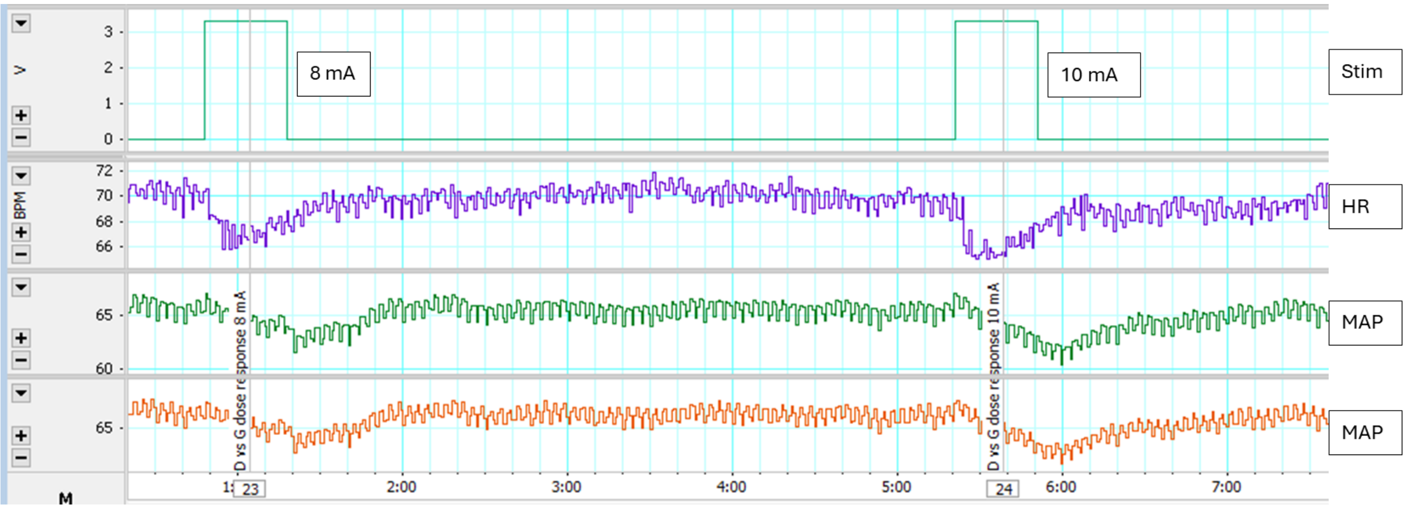

Subject 9

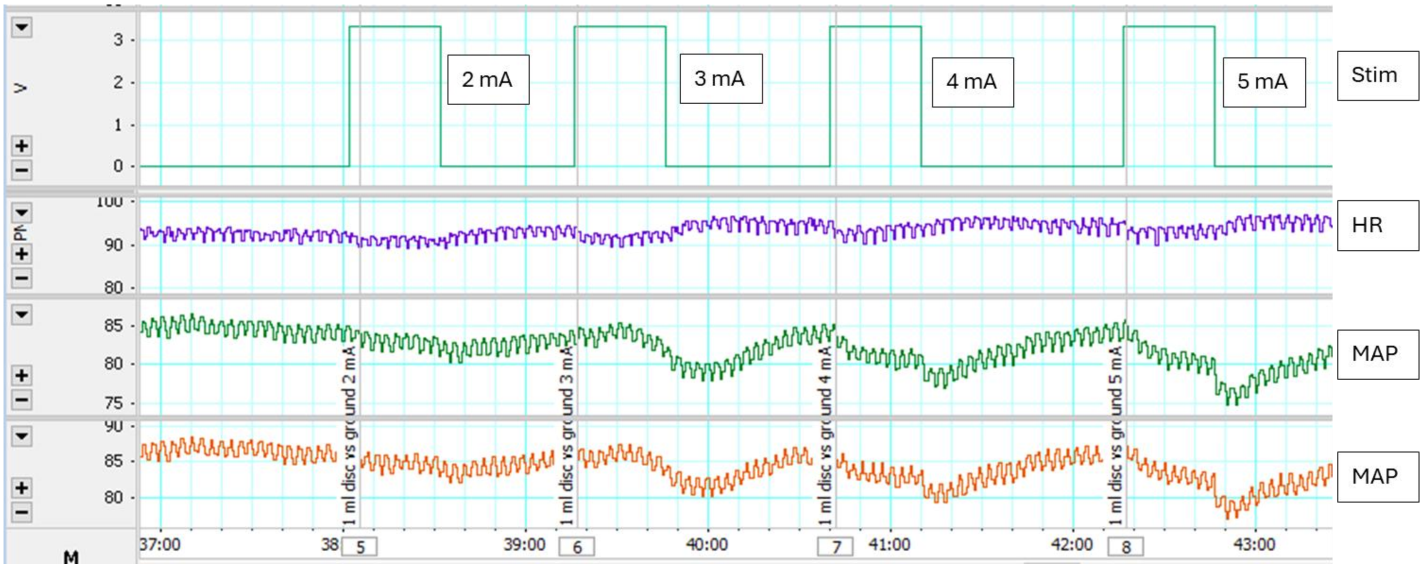

Subject 11

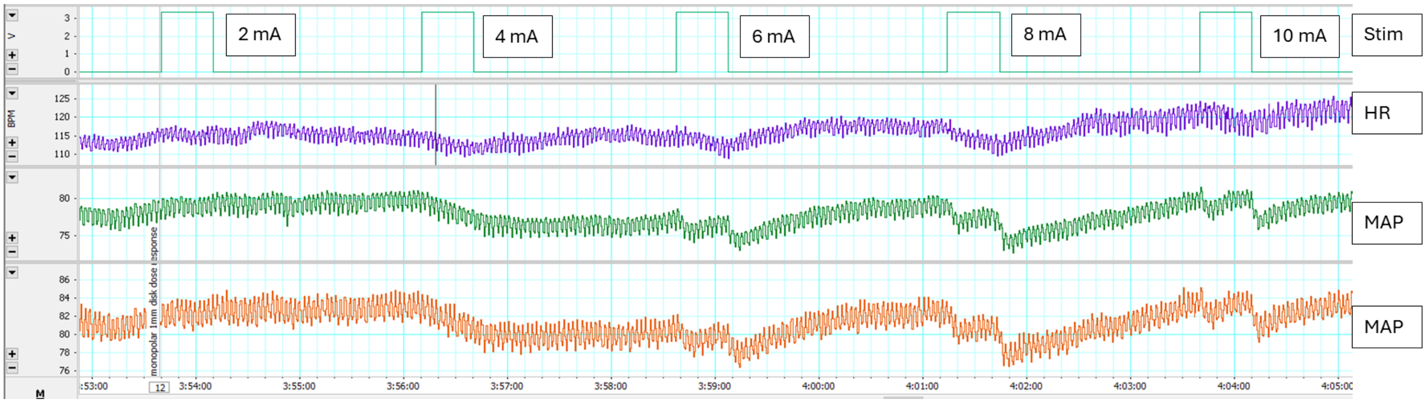

Supplemental Figure 8: Blood pressure drops from additional subjects from cohort collected during dose response curve stimulation.

Outlier from EMG Dose response curve analysis

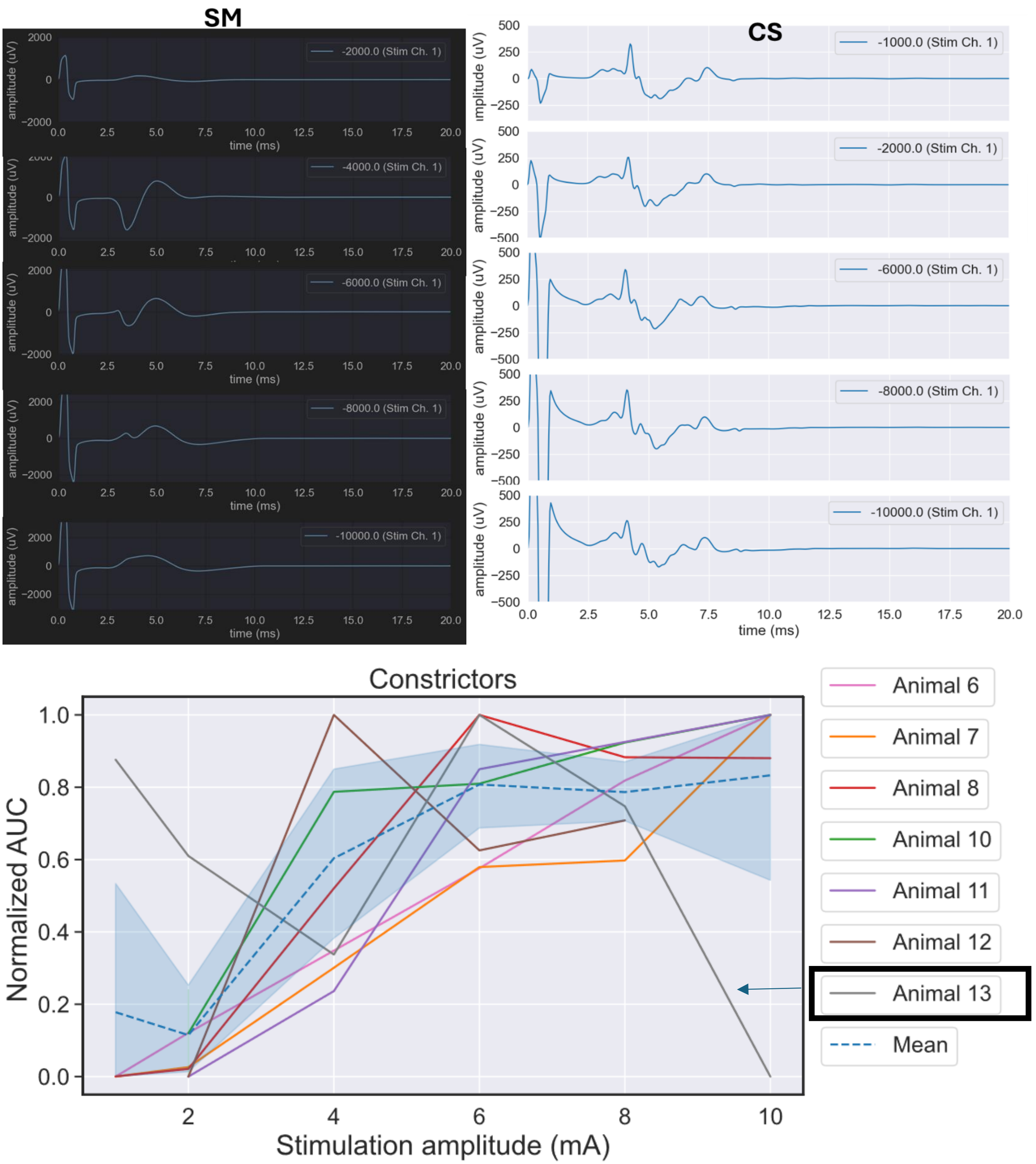

Supplemental Figure 9: Subject 13 had atypical of EMG dose response curve wherein higher amplitudes evoked smaller EMG than lower intensities. There could be multiple reasons for this including instrumentation error in the EMG wires where a large muscle contraction caused the wires to be lose, change in the fluid/edema in the pocket which changed the activation pathway, improper placement of EMG electrodes to record the evoked signal accurately or muscle fatigue.

Cohort EMG recordings for selecting activation threshold per muscle

Subject 6

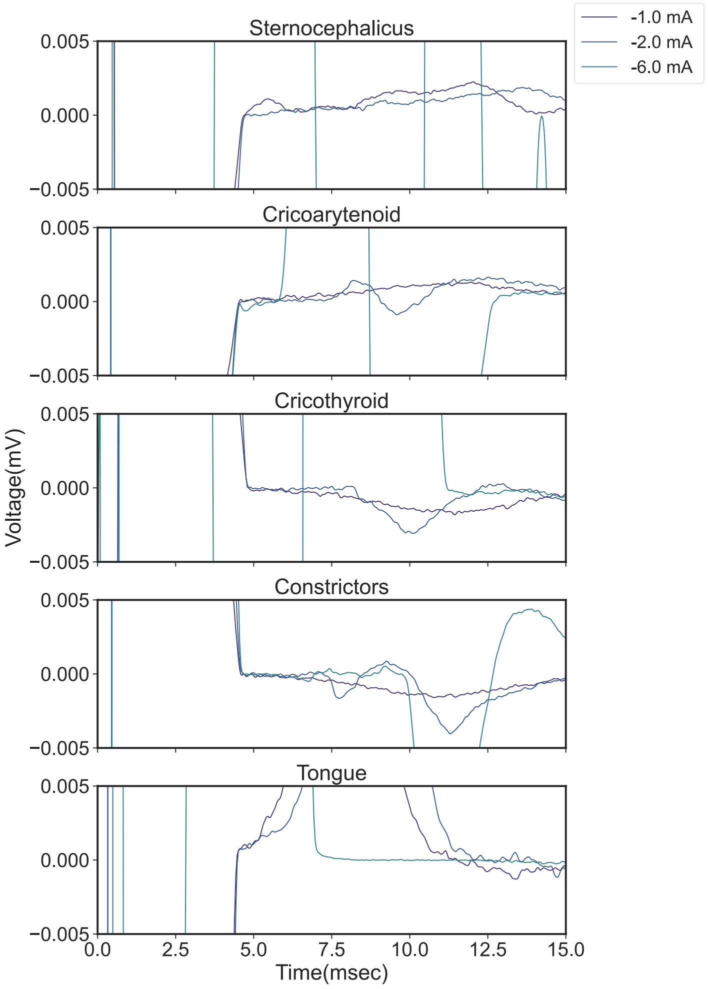

Subject 7

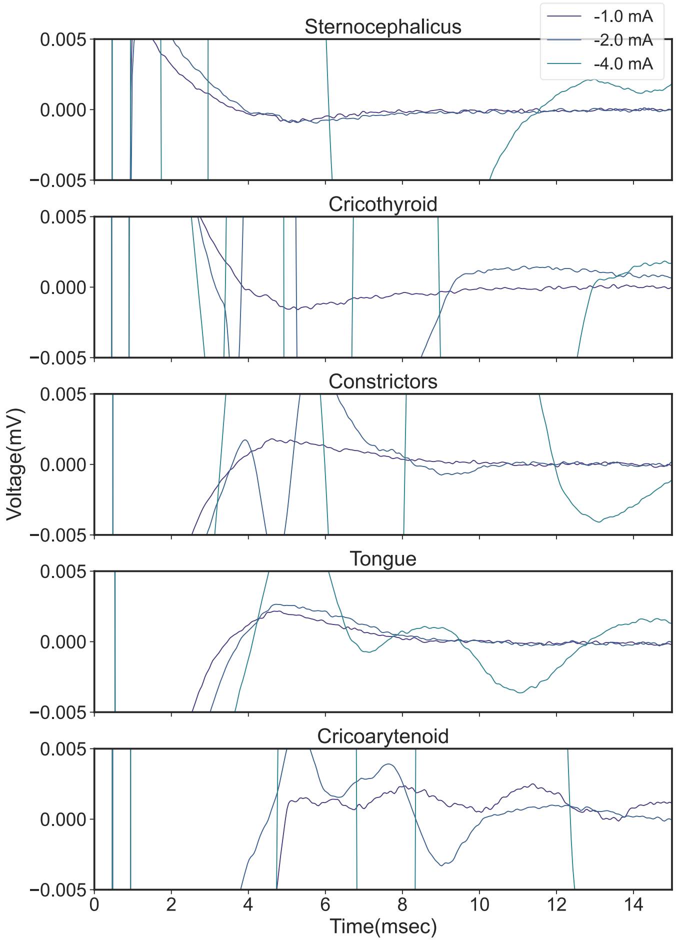

Subject 9

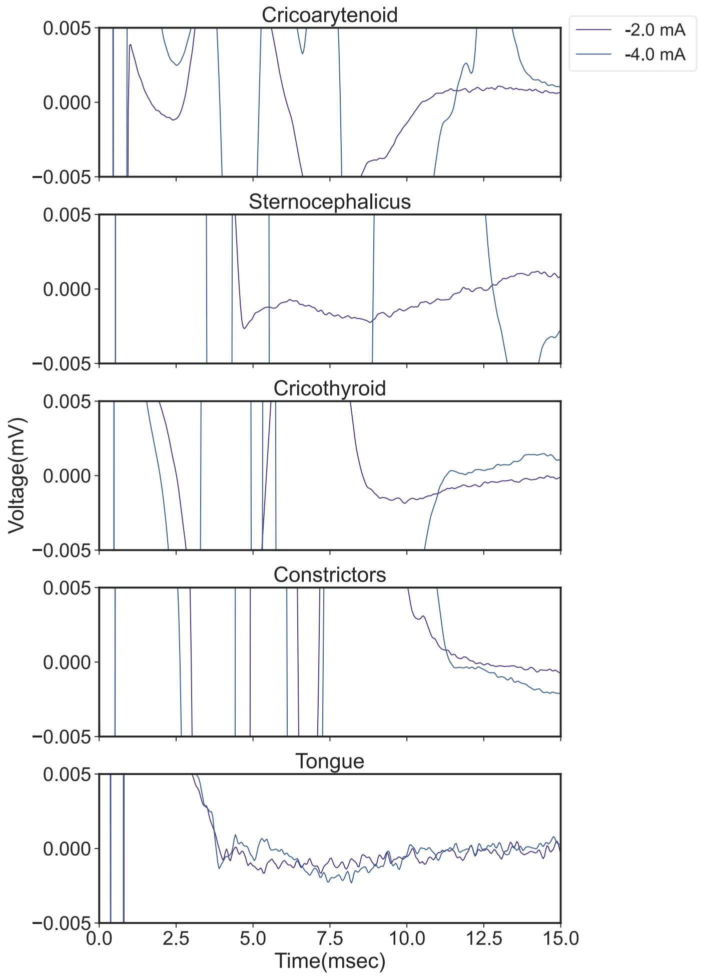

Subject 10

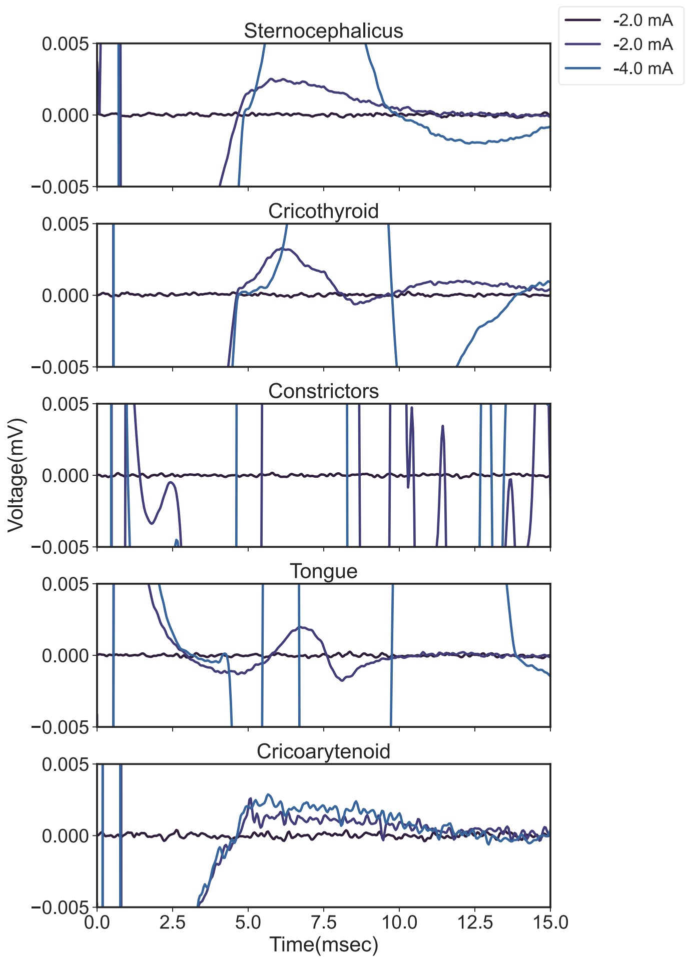

Subject 11

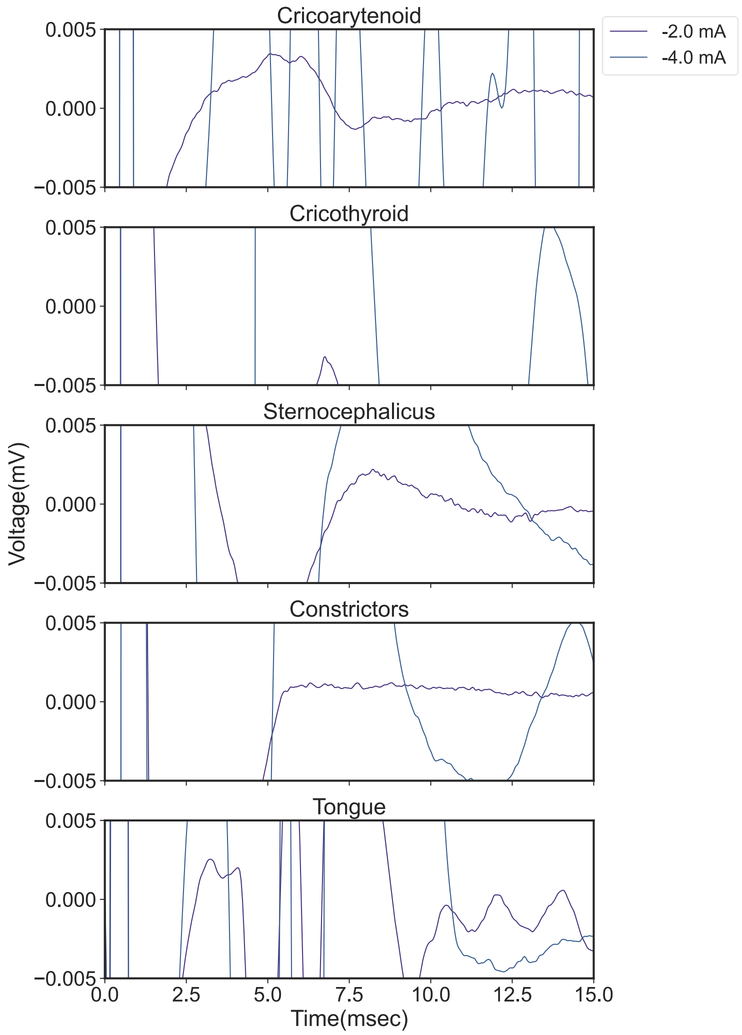
Cricoarytenoid
Cricothyroid
Sternocephalicus
Constrictors
Tongue
-2.0 mA
-4.0 mA
Voltage(mV)
Time(msec)
0.005
0.000
−0.005
0.0
2.5
5.0
7.5
10.0
12.5
15.0

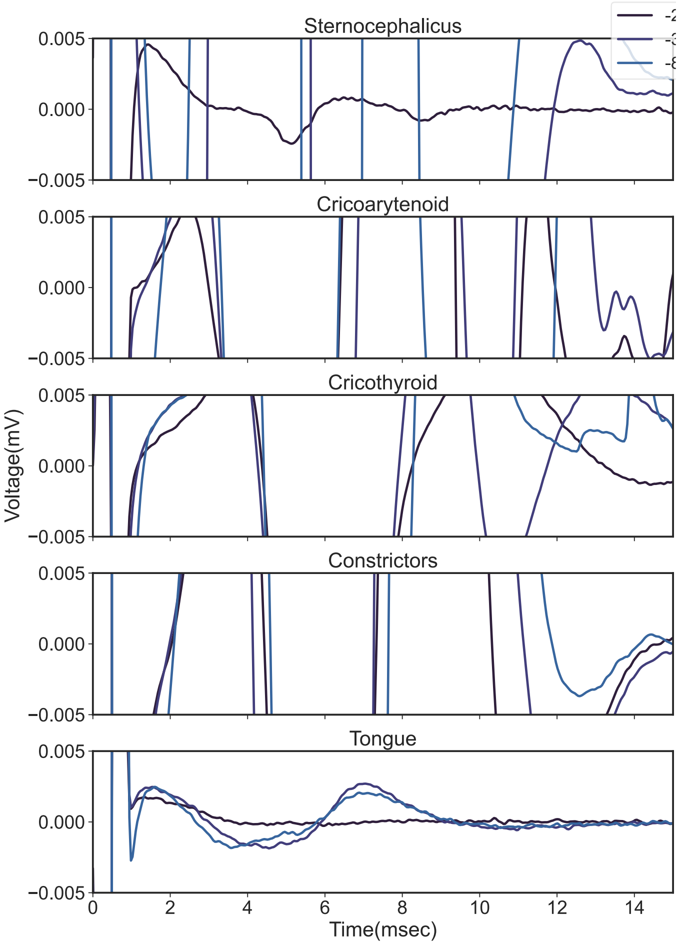
Sternocephalicus
Cricoarytenoid
Cricothyroid
Constrictors
Tongue
Voltage(mV)
Time(msec)
0.005
0.000
−0.005
0
2
4
6
8
10
12
14

Subject 13

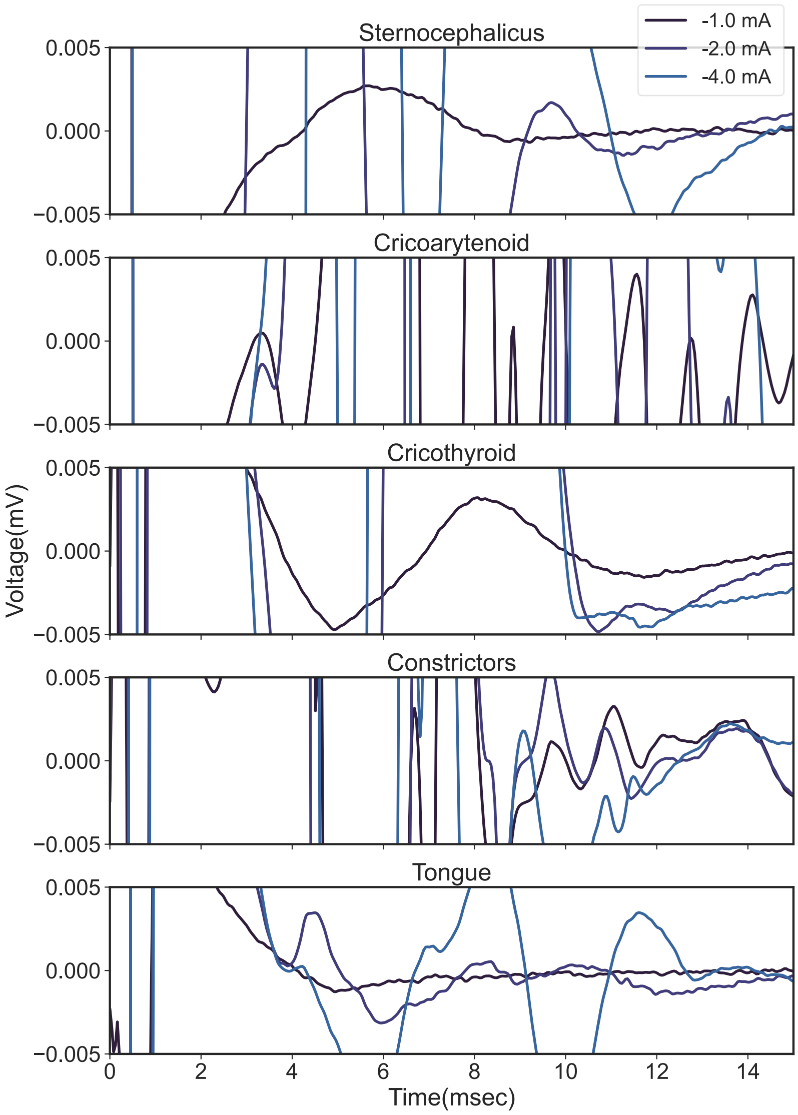

Supplemental Figure 10: Cohort EMG recordings to identify activation thresholds for different muscle groups. If the EMG signal of a particular muscle crossed the amplitude window of 10 uV pk-pk, that stimulation amplitude was noted as the activation threshold for that particular muscle group.
